# An efficient and accurate interpolation method for parametric curve machining

**DOI:** 10.1038/s41598-022-20018-9

**Published:** 2022-09-26

**Authors:** Juan Wei, Chao Sun, Xue-jing Zhang, Er-jie Wang, Deify Law

**Affiliations:** 1 College of Mechanical Engineering, Xi’an University of Science and Technology, Xi’an, 710054 China; 2 Shaanxi Provincial Key Laboratory of Mine Electromechanical Equipment Intelligent Monitoring, Xi’an, 710054 China; 3 Department of Mechanical Engineering, California State University, Fresno, Fresno, CA 93740-8030 USA

**Keywords:** Mechanical engineering, Design, synthesis and processing

## Abstract

A subsection interpolation method based on the curve curvature threshold is proposed to resolve the incompatible problem of machining accuracy and machining efficiency in parametric curve machining. In the pre-interpolation stage, the curve curvature threshold is calculated based on geometric and kinematic constraints. The subsection interpolation key points and their nominal velocities are then determined from the curvature threshold points and the start and end points of the curve, and the arc length of each subsegment can be calculated based on the adaptive Simpson method. As a result, the S-type speed planning algorithm and the bidirectional speed scanning algorithm are used to update and realize the global speed curve to reduce the speed fluctuation. In the real-time interpolation stage, the curve interpolation parameters are calculated using the parametric modified second-order Runge–Kutta method, which could improve the interpolation accuracy significantly and also shorten the interpolation time. Finally, it is found using numerical cases that the proposed method can smooth the overall interpolation speed, reduce the speed fluctuation effectively and improve the real-time performance of the interpolation.

## Introduction

The Non-Uniform Rational B-Spline (NURBS) has good local control ability and shape expression ability, and has been widely used in the construction of free curves and surfaces^1^. The interpolation technology based on NURBS can directly interpolate parametric curves without separating the curves into a large number of straight lines and arcs, thus avoiding frequent acceleration and deceleration in the processing process. It would improve greatly the machining accuracy and efficiency. With the increasing demand for machining complex surface parts, complex surface modeling and machining technology based on NURBS technology has become the key technology for achieving high-efficiency precision machining and has attracted more and more attention from scholars. Wei et al.^2^ studied the integral impeller modeling and tool path planning based on NURBS curve and surface, and realized the design and processing of complex surface parts based on unified NURBS parameters, but its processing process depended on high-grade NC machine tools with NURBS interpolation function.

At present, the research on NURBS interpolation at home and abroad mainly focuses on two aspects: speed planning algorithm and real-time spline interpolation parameter calculation. In numerical control (NC) machining, the tool moves along the given path of the parameter curve, and due to the kinematic and geometric constraints, the preset speed planning method can ensure the smooth splicing of multiple speed curves. Wang et al.^3–5^ used constant feed speed to interpolate the parameter curve, the method is conducive to the stability of the processing process for the curve with little change in curvature, but for the parameter curve with variable curvature, the processing accuracy and processing efficiency cannot be considered. Nam et al.^6–9^ proposed an algorithm that self-adaptive S-type acceleration/deceleration planning for meeting the kinematic constraints of the machine tool to realize the smooth transition of feed speed, so this method is one of the most widely used speed planning algorithms in the field of NC machining^10–14^. Lee et al.^15^ and Wang et al.^16^ put forward the speed planning method of trigonometric function to realize the smooth change of acceleration and jerk, but its processing process only reaches the extreme value of motion parameters at individual times, can not make full use of machine tools, and the motion efficiency is low. Liu et al.^17^ added positive and negative speed verification points in the forward-looking interpolation module based on S-type acceleration and deceleration planning, and determined whether to call the reverse interpolation verification point interpolation according to the speed judgment conditions in the real-time interpolation stage. This method can effectively improve interpolation efficiency. Zhang et al.^18^ used five B-sample curves to generate a toolpath with smooth curvature based on theoretical feed rate constraints bounded by axis acceleration and impact. Chen et al.^19^ proposed a five-polynomial acceleration/deceleration control algorithm, which is able to achieve flexible control of acceleration. LI et al.^20^ used Sigmoid function feedrate profile, which is more concise compared with the polynomial profile and more efficient compared with the trigonometric profile.

The relationship between NURBS curve arc length and parameters is nonlinear, so it is necessary to express the relationship by numerical method. When interpolating the curve, it is necessary to calculate the curve parameters corresponding to the interpolation point of each interpolation cycle according to the preset speed programming method. Usually, the interpolation point parameters are calculated by the direct method or iterative method, and the direct method mainly uses Taylor series expansion. Shipitalni et al.^21^ used the first-order Taylor expansion method to calculate the parameters of each interpolation point for the first time, but the interpolation error was large because of abandoning the higher-order term. Yang et al.^22^ adopted the second-order Taylor expansion, which improved the interpolation accuracy. However, the introduction of the second-order derivative had a large amount of computation, which affected the real-time performance, and Taylor expansion inevitably introduced truncation errors. Han et al.^23^ used the Runge–Kutta method to calculate interpolation parameters. The accuracy of this method is relatively high, but the first derivative must be solved four times every time. Peng et al.^24^ and Ji et al.^25^ used Adams–Bashforth and Adams–Moultou methods to calculate interpolation parameters in the real-time interpolation stage, which can consider both interpolation accuracy and interpolation efficiency. The interpolation point parameter iteration method refers mainly to the "estimation–correction" method, which obtains the deviation between actual and ideal parameters through the estimation, and then corrects the deviation to a given range through repeated iteration. Zhao et al.^26^ proposed an interpolation parameter calculation method with arc length correction and feedback correction, which can improve interpolation efficiency and accuracy. Ni et al.^27^ proposed a quintic polynomial prediction algorithm, and estimated the target arc length in the second-order Taylor expansion to improve the calculation accuracy and iterative convergence speed. The iteration method needs to be repeated in each interpolation cycle, and the number of iterations is not fixed, which affects the real-time performance of interpolation.

In this paper, the data sampling interpolation algorithm is used to process the parameter curve in two stages: pre-interpolation and real-time interpolation. In the pre-interpolation phase, the minimum value of the chord error constraint and the velocity, acceleration and acceleration constraints are used as the curve curvature threshold. Setting the points on the curve where the curvature equals and exceeds the curvature threshold as key points, dividing the curve into curve segments based on the key points and calculating the nominal velocity at each key point according to the constraints. The S-shaped velocity planning method is used to achieve continuous and bounded acceleration within each curve segment; at each key point the velocity two-way scanning method is used to achieve continuous and bounded acceleration in adjacent curve segments. At the same time, in order to further improve the interpolation calculation accuracy and efficiency, a parametric modified second-order Runge–Kutta method is proposed in the real-time interpolation stage, which uses only three first-order derivative calculations after introducing the parameter correction values to obtain a high calculation accuracy, avoiding the complex calculation volume of traditional interpolation algorithms. The combination of the proposed velocity planning method and the parametric interpolation algorithm can also reduce the amount of interpolation calculations while ensuring interpolation accuracy and improving interpolation real time performance. Finally, the validity of the proposed interpolation algorithm is discussed by simulation.

## NURBS curve interpolation

### NURBS curve definition

The general expression of the NURBS curve^28^:

1 $$C(u) = \frac{{\sum\limits_{i = 0}^{n} {N_{i,k} (u)w_{i} P_{i} } }}{{\sum\limits_{i = 0}^{n} {N_{i,k} (u)w_{i} } }},\quad 0 \le u \le 1.$$

where $k$ is the number of times of the NURBS curves, $u$ is the curve parameter, $P_{i}$ is the control vertex, and these points can form a NURBS curve control polygon, $\omega_{i}$ represents the weight corresponding to the control vertex, $N_{i,k} (u)$ is the *i*th k-degree B-spline basis function defined on the non-periodic and non-uniform node vector U. Defined by the De-Boor recursion as:

2 $$\begin{aligned} & N_{i,0} = \left\{ {\begin{array}{*{20}l} {1,\quad u_{i} \le u \le u_{i + 1} } \hfill \\ {{0,}\quad {\text{otherwise}}} \hfill \\ \end{array} } \right. \\ & N_{i,k} (u) = \frac{{u - u_{i} }}{{u_{i + k} - u_{i} }}N_{i,k - 1} (u) + \frac{{u_{i + k + 1} - u}}{{u_{i + k + 1} - u_{i + 1} }}N_{i + 1,k - 1} (u). \\ \end{aligned}$$

where $U = \left\{ {\underbrace {0, \ldots 0,}_{k + 1}u_{k + 1} , \ldots ,u_{n} ,\underbrace {1, \ldots ,1}_{k + 1}} \right\}$ is a monotonous sequence of reduced real numbers.

### NURBS interpolation parameters calculation

Since there is no exact precise analytical relationship between curve parameters and arc length, it is necessary to select an appropriate numerical interpolation method to obtain curve parameters, and then generate interpolation position instructions. In this paper, a parameter-modified second-order Runge–Kutta interpolation algorithm is used to calculate real-time interpolation parameters. Firstly, the second-order Runge–Kutta method can be used to calculate the initial value $\tilde{u}_{i + 1}$ of the interpolation parameters in the next interpolation cycle, which is based on the interpolation speed $V(u_{i} )$ and interpolation parameter $u_{i}$ in the current interpolation cycle:

3 $$\tilde{u}_{i + 1} = u_{i} + \frac{T}{2}(K_{1} + K_{2} ).$$

where $T$ is the interpolation period, $C^{\prime}(u_{i} )$ is the first order derivative of the NURBS curve, $K_{1}$ and $K_{2}$ are given by

4 $$K_{1} = \frac{{V(u_{i} )}}{{\left\| {C^{\prime}(u_{i} )} \right\|}}.$$

5 $$K_{2} = \frac{{V(u_{i} )}}{{\left\| {C^{\prime}(u_{i} + TK_{1} )} \right\|}}.$$

According to Eq. (3), the initial parameter value of the new interpolation point can be obtained, and then the parameter modification value $\Delta u_{i + 1}$ can be calculated. To minimize the speed fluctuation, the actual interpolation displacement in the interpolation period should be equal to the ideal interpolation displacement, that is, the following equation should be satisfied:

6 $$\left\| {C\left( {\tilde{u}_{i + 1} + \Delta u_{i + 1} } \right) - C\left( {u_{i} } \right)} \right\| = V(u_{i} )T.$$

The first-order Taylor series expansion of the NURBS curve parameter equation $C(u)$ at the initial parameter value $\tilde{u}_{i + 1}$ can be obtained as:

7 $$C\left( {\tilde{u}_{i + 1} + \Delta u_{i + 1} } \right) = C\left( {\tilde{u}_{i + 1} } \right) + C^{\prime}\left( {\tilde{u}_{i + 1} } \right)\Delta u_{i + 1} .$$

Substituting Eq. (7) into Eq. (6) yields

8 $$\left\| {C\left( {\tilde{u}_{i + 1} } \right) + C^{\prime}\left( {\tilde{u}_{i + 1} } \right)\Delta u_{i + 1} - C\left( {u_{i} } \right)} \right\|^{2} = V^{2} \left( {u_{i} } \right)T^{2} .$$

With the help of the previous relation, Eq. (8) can then be written as

9 $$c_{1} \Delta u_{i + 1}^{2} + c_{2} \Delta u_{i + 1} + c_{3} = 0.$$

In the Eq. (9), the coefficients $c_{1}$, $c_{2}$ and $c_{3}$ are defined as

10 $$\begin{aligned} c_{1} & = \left\| {C^{\prime}(\tilde{u}_{i + 1} )} \right\|^{2} \\ c_{2} & = 2C^{\prime}\left( {\tilde{u}_{i + 1} } \right)^{T} \cdot \left( {C\left( {\tilde{u}_{i + 1} } \right) - C\left( {u_{i} } \right)} \right) \\ c_{3} & = \left\| {C\left( {\tilde{u}_{i + 1} } \right) - C\left( {u_{i} } \right)} \right\|^{2} - V^{2} \left( {u_{i} } \right)T^{2} . \\ \end{aligned}$$

Two roots $\Delta u_{i + 1,1}$ and $\Delta u_{i + 1,2}$ of the parameter correction value $\Delta u_{i + 1}$ can be obtained by solving the quadratic Eq. (9):

11 $$\begin{aligned} \Delta u_{i + 1,1} & = \frac{{ - c_{2} + \sqrt {c_{2}^{2} - 4c_{1} c_{3} } }}{{2c_{1} }} \\ \Delta u_{i + 1,2} & = \frac{{ - c_{2} - \sqrt {c_{2}^{2} - 4c_{1} c_{3} } }}{{2c_{1} }}. \\ \end{aligned}$$

Since the Runge–Kutta method can achieve second-order accuracy, the coefficient $c_{3} \approx 0$ holds. Therefore, $\Delta u_{i + 1,1} \approx 0$, $\Delta u_{i + 1,2} \approx - \frac{{c_{2} }}{{c_{1} }}$. To meet the stability requirements of the interpolation algorithm, if Eq. (9) has no real solution, the parameter correction value is set to 0, if Eq. (9) has a real solution, the smaller solution is taken as the parameter correction value. That is, the parameter modification value $\Delta u_{i + 1}$ can be calculated as:

12 $$\Delta u_{i + 1} = \left\{ {\begin{array}{*{20}l} {\frac{{ - c_{2} + \sqrt {c_{2}^{2} - 4c_{1} c_{3} } }}{{2c_{1} }},} & {c_{2}^{2} - 4c_{1} c_{3} \ge 0} \\ 0 & {c_{2}^{2} - 4c_{1} c_{3} < 0} \\ \end{array} } \right..$$

Therefore, the parameter of the next interpolation point of the curve can be obtained by adding the initial parameter value and the parameter correction value:

13 $$u_{i + 1} = \tilde{u}_{i + 1} + \Delta u_{i + 1} .$$

#### Remark

*According to the above calculation process of interpolation parameters, it can be found clearly that the parametric modified second-order Runge–Kutta interpolation algorithm developed here only performs three first-order derivative calculations and does not carry out higher-order derivative operations. As a result, the calculation accuracy can be improved on the premise of reducing the cost of computations.*

## Acceleration and deceleration control method

The real-time control and planning of the feed speed are the two key factors to realize the high precision and high efficiency of CNC machining. The flexible acceleration and deceleration control can make the tool and machine tool parts run smoothly in the machining process, without the phenomenon of shock, impact, out-of-step, and so on.

**Figure 1:**
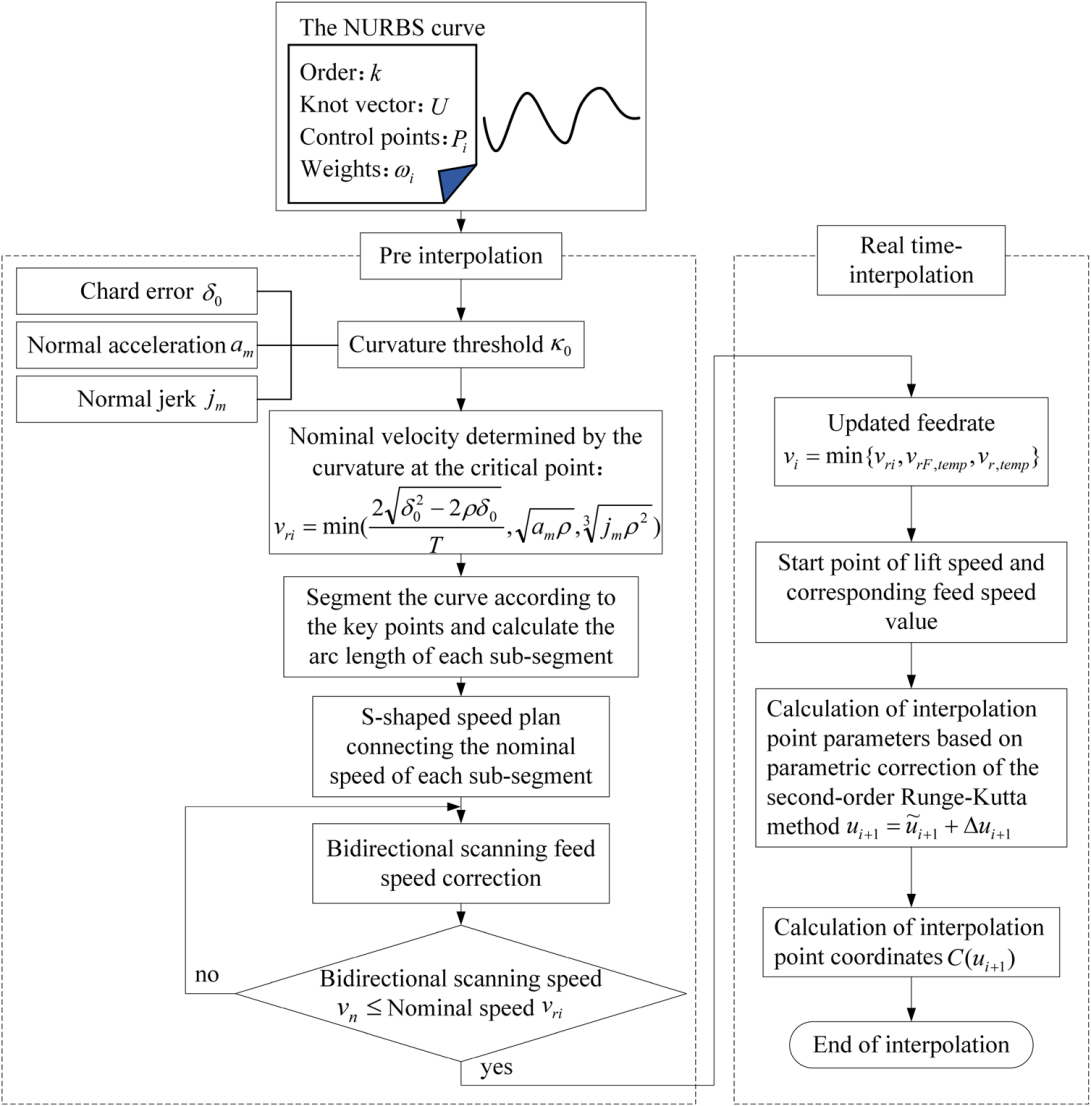
The overall process of the interpolation method.

In this paper, the S-type curve acceleration/deceleration planning method is chosen to realize curve interpolation speed control, and the parameter-modified second-order Runge–Kutta method is adopted to calculate interpolation point parameters to realize real-time interpolation. The interpolation process is shown in Fig. 1.

In the pre-processing stage, through the bow of conditions such as high error and normal acceleration calculated curvature threshold, and then find out the point of maximum curvature and curvature point of the threshold value is determined as the key point is not less than, according to the key points of NURBS curve is divided into several NURBS period, each section of the arc length calculation, based on S-type velocity planning algorithm smooth connecting paragraphs beginning and end of the curve of the nominal speed, The final interpolation velocity curve is obtained by bidirectional velocity scanning. Finally, the second-order Runge–Kutta interpolation algorithm with parameter modification proposed in Section 1.2 is used to obtain real-time position instructions according to the planned velocity curve to complete the interpolation.

### Interpolation preprocessing

**Figure 2:**
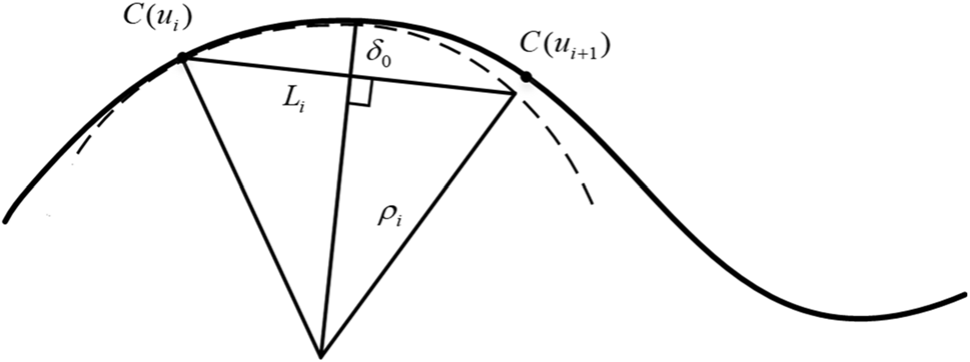
Schematic diagram of chord error.

NURBS curve interpolation uses chord length to approximate arc length, so chord error will be generated, as shown in Fig. 2. The close circular arc is commonly used to approximate the curve at the current interpolation point $C(u_{i} )$, and the nominal velocity $v_{ui}$ at the point $C(u_{i} )$ can be obtained from the close circular radius $\rho_{i}$ of curvature and the chord error $\delta_{i}$ as follows^29^:

14 $$v_{ui} \le \frac{{2\sqrt {\delta_{i}^{2} - 2\rho_{i} \delta_{i} } }}{T}.$$

Similarly, after considering the normal acceleration and normal jerk, the nominal velocity $v_{ri}$ can be given by Eq. (15)^30^.

15 $$v_{ri} = \min \left( {\frac{{2\sqrt {\delta_{0}^{2} - 2\rho \delta_{0} } }}{T},\sqrt {a_{m} \rho } ,\sqrt[3]{{j_{m} \rho^{2} }}} \right).$$

where $\delta_{0}$ is the set maximum chord error, $T$ is the interpolation period, $a_{m}$ and $j_{m}$ are the maximum acceleration and maximum jerk allowed by the machine.

Denote $\kappa = \frac{1}{\rho }$, when $v_{ri}$ is equal to maximum federate $v_{m}$, the curvature threshold of the parameter curve $\kappa_{0}$ can be obtained as shown in Eq. (16).

16 $$\kappa_{0} = \min \left( {\frac{{8\delta_{0} }}{{v_{m}^{2} T^{2} + 4\delta_{0}^{2} }},\frac{{a_{m} }}{{v_{m}^{2} }},\sqrt {\frac{{j_{m} }}{{v_{m}^{3} }}} } \right).$$

**Figure 3:**
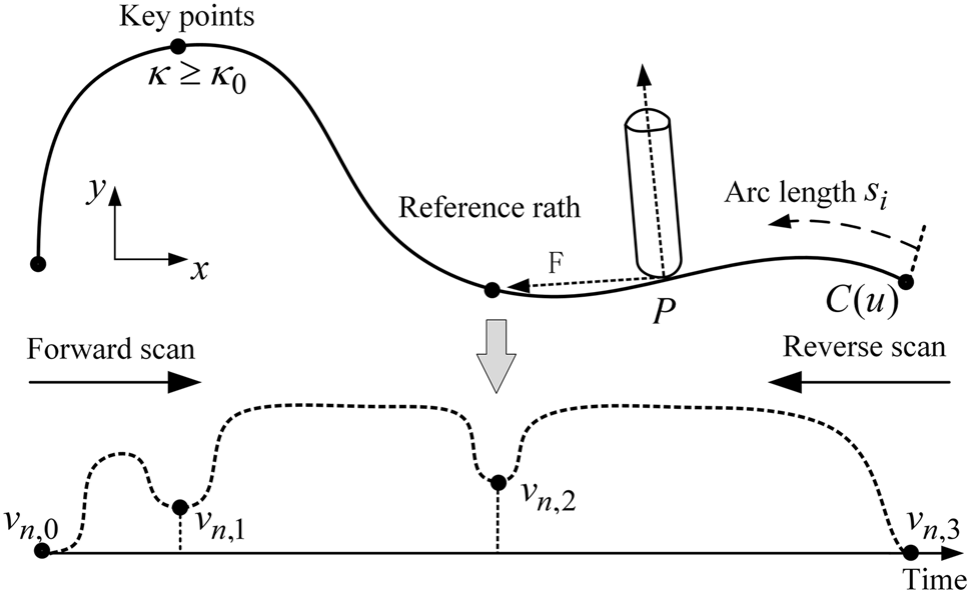
Flowchart of acceleration and deceleration planning.

After the curve curvature threshold $\kappa_{0}$ is obtained, all the curvature maximum points on the curve are obtained. If the curvature at this curvature maximum point is also satisfied that *k* is greater than or equal to $\kappa_{0}$, these points are determined as the key points. The resulting key points including the start and end points of the curve are all the key points, as shown in Fig. 3. Then, set the nominal speed at the first and last key points to zero and obtain the nominal speed $v_{ri}$ of each key point except the first and last key points from Eq. (15). The curve is divided into several sections at key points, and the endpoint velocity of each sub-section is the nominal velocity $v_{ri}$. Then, the arc length S of each sub-section is determined by using the self-adaptive Simpson method. Finally, the velocity function of the starting and ending boundary velocities is determined according to the S-type acceleration and deceleration method and the bidirectional velocity scanning algorithm.

### S-type acceleration and deceleration control

The NURBS curve is divided into several sub-segments, and the starting and ending velocities of each sub-segment are connected according to the preset acceleration and deceleration method. To realize high quality and efficient machining, the S-type acceleration and deceleration control algorithm is given below.

**Figure 4:**
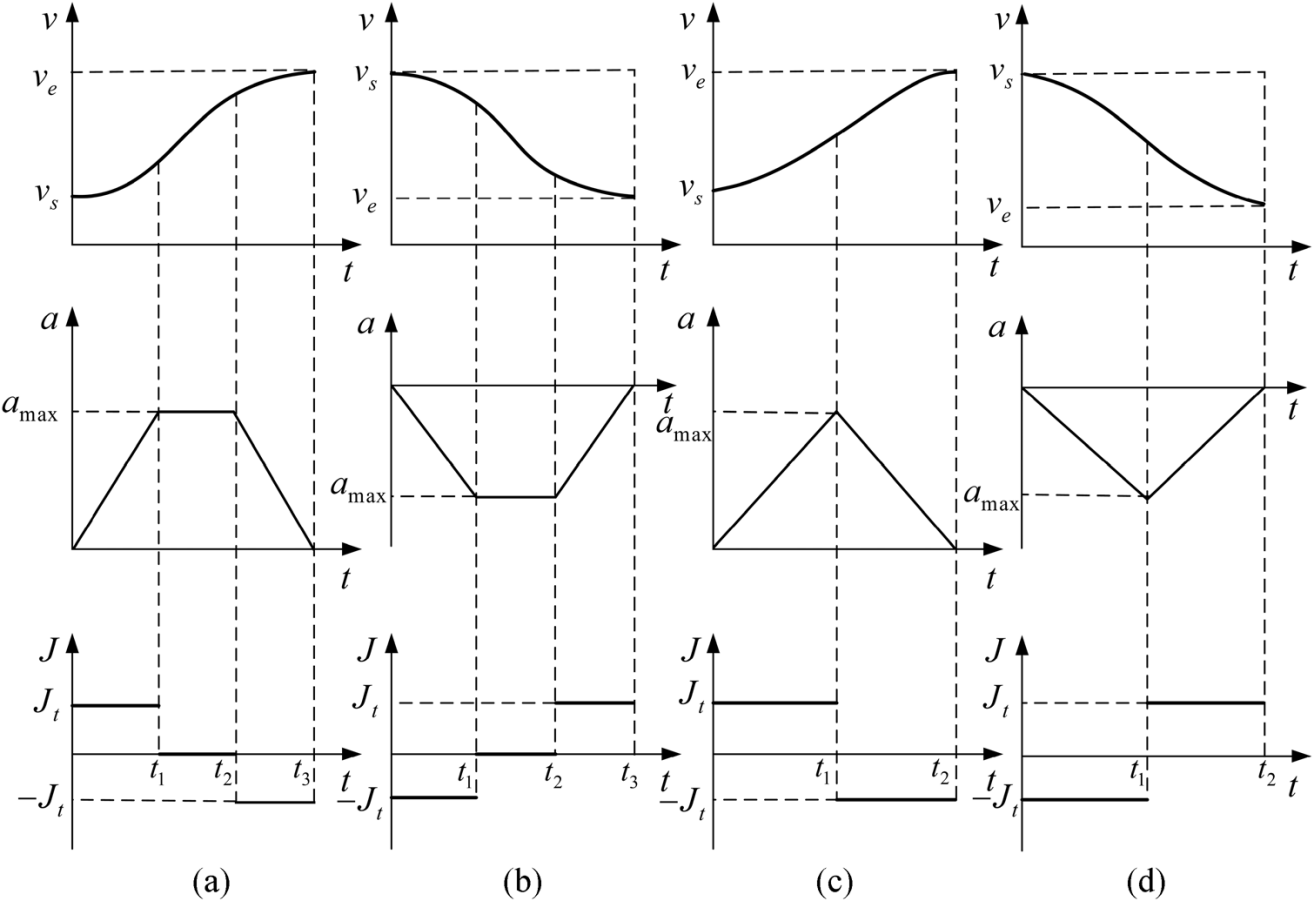
S-type acceleration and deceleration planning of (**a**) Elevated acceleration-constant rate-reduced acceleration, (**b**) Reduced acceleration-constant rate-reduced deceleration, (**c**) Elevated acceleration-elevated deceleration, (**d**) Reduced acceleration-reduced deceleration.

When the maximum values of acceleration and jerk are $a_{\max }$ and $J_{t}$, and the initial and ending velocities are $v_{s}$ and $v_{e}$, the s-shaped acceleration and deceleration planning outline are shown in Fig. 4.

In order to ensure acceleration and deceleration efficiency, the paths of each sub-section are connected according to the arc length *s*, starting speed $v_{s}$ and ending speed $v_{e}$ of each section of the curve. The four common speed curves are shown in Fig. 4. When the sub-arc length is long, the velocity curve is of the type of elevated acceleration-constant rate-reduced acceleration (reduced acceleration-constant rate-reduced deceleration). When the sub-arc length is short, the velocity curve is of the type of elevated acceleration-elevated deceleration (reduced acceleration-reduced deceleration).

Under the constraints of maximum acceleration $a_{\max }$ and jerk $J_{t}$, the time of elevated acceleration (reduced acceleration) $t_{1}$, the time of constant rate $t_{2}$, the time of reduced acceleration (reduced deceleration) $t_{3}$ of S-type acceleration and deceleration planning is expressed as follows:

17 $$\begin{aligned} t_{1} & = \left\{ {\begin{array}{*{20}l} {\frac{{a_{\max } }}{{J_{t} }},} & {\left| {v_{e} - v_{s} } \right| > \frac{{a_{\max }^{2} }}{{J_{t} }}} \\ {\sqrt {\frac{{v_{e} - v_{s} }}{{J_{t} }},} } & {\left| {v_{e} - v_{s} } \right| \le \frac{{a_{\max }^{2} }}{{J_{t} }}} \\ \end{array} } \right. \\ t_{2} & = \left\{ {\begin{array}{*{20}l} {\frac{{v_{e} - v_{s} - a_{\max }^{2} /J_{t} }}{{a_{\max } }},} & {\left| {v_{e} - v_{s} } \right| > \frac{{a_{\max }^{2} }}{{J_{t} }}} \\ {0,} & {\left| {v_{e} - v_{s} } \right| \le \frac{{a_{\max }^{2} }}{{J_{t} }}} \\ \end{array} } \right. \\ t_{3} & = t_{1} . \\ \end{aligned}$$

Suppose that the ultimate acceleration $a_{\max }$ in the process of acceleration/deceleration is equal to $J_{t} t_{1}$, then the relationship between velocity $v$ and time $t$ in the process of acceleration/deceleration can be expressed as:

18 $$v(t) = \left\{ {\begin{array}{*{20}l} {v_{s} \pm \frac{1}{2}J_{t}^{2} ,} & {0 \le t < t_{1} } \\ {v_{1} \pm a_{\max } (t - t_{1} ),v_{1} = v_{s} \pm \frac{1}{2}J_{t} t_{1}^{2} ,} & {t_{1} \le t < t_{1} } \\ {v_{2} \pm a_{\max } (t - t_{1} - t_{2} ) \mp \frac{1}{2}J_{t} (t - t_{1} - t_{2} )^{2} ,} & {t_{1} + t_{2} \le t_{1} + t_{2} + t_{3} } \\ {v_{2} = v_{1} \pm a_{\max } t_{2} } &{} \\ \end{array} } \right..$$

where “$\pm$” is “$+$” and “$-$” in the process of speed increase; “$\mp$” is, however, “$-$” and “$+$” in the process of speed decrease.

Therefore, the relationship between displacement $s$ and time $t$ in the process of acceleration/deceleration is:

19 $$s(t) = \left\{ {\begin{array}{*{20}l} {v_{s} \pm \frac{1}{6}J_{t} t^{3} ,} & {0 \le t < t_{1} } \\ {s_{1} + v_{1} (t - t_{1} ) \pm \frac{1}{2}a_{\max } (t - t_{1} )^{2} ,s_{1} = v_{s} t_{1} \pm \frac{1}{6}J_{t} t_{1}^{3} ,} & {t_{1} \le t < t_{1} + t_{2} } \\ {s_{2} + v_{2} (t - t_{1} - t_{2} ) \pm \frac{1}{2}a_{\max } (t - t_{1} - t_{2} )^{2} \mp \frac{1}{6}J_{t} (t - t_{1} - t_{2} )^{3} ,} & {} \\ {s_{2} = s_{1} + v_{1} t_{2} \pm \frac{1}{2}a_{\max } t_{2}^{2} ,} & {t_{1} + t_{2} \le t \le t_{1} + t_{2} + t_{3} } \\ \end{array} } \right..$$

According to the above S-type acceleration and deceleration process, the total displacement $s$ of the acceleration/deceleration movement from the starting speed $v_{s}$ to the ending speed $v_{e}$ is:

20 $$s = \left\{ {\begin{array}{*{20}l} {v_{s} \left( {t_{1} + t_{2} + t_{3} } \right) + \frac{1}{2}J_{t} t_{1}^{2} \left( {t_{2} + t_{3} } \right) + \frac{1}{2}a_{\max } \left( {t_{2}^{2} + t_{3}^{2} } \right) + a_{\max } t_{2} t_{3} ,\quad v_{s} < v_{e} } \\ {v_{s} \left( {t_{1} + t_{2} + t_{3} } \right) - \frac{1}{2}J_{t} t_{1}^{2} \left( {t_{2} + t_{3} } \right) - \frac{1}{2}a_{\max } \left( {t_{2}^{2} + t_{3}^{2} } \right) - a_{\max } t_{2} t_{3} ,\quad v_{s} > v_{e} } \\ \end{array} } \right..$$

### Key point speed update

As the exact solution of arc length of each curve cannot be obtained, numerical methods are generally used to calculate the approximate value of arc length. In order to meet the calculation accuracy and give consideration to the numerical calculation efficiency, the adaptive Simpson method^31^ is used to calculate arc length here $s(u_{i} ,u_{i + 1} )$:

21 $$s\left( {u_{i} ,u_{i + 1} } \right) = \frac{{\left( {u_{i + 1} - u_{i} } \right)}}{6}\left[ {C^{\prime}\left( {u_{i} } \right) + 4C^{\prime}\left( {\frac{{u_{i + 1} + u_{i} }}{2}} \right) + C^{\prime}\left( {u_{i + 1} } \right)} \right].$$

where $C^{\prime}(u_{i} )$ and $C^{\prime}(u_{i + 1} )$ are the first derivatives of the curve at parameters $u_{i}$ and $u_{i + 1}$.

**Figure 5:**
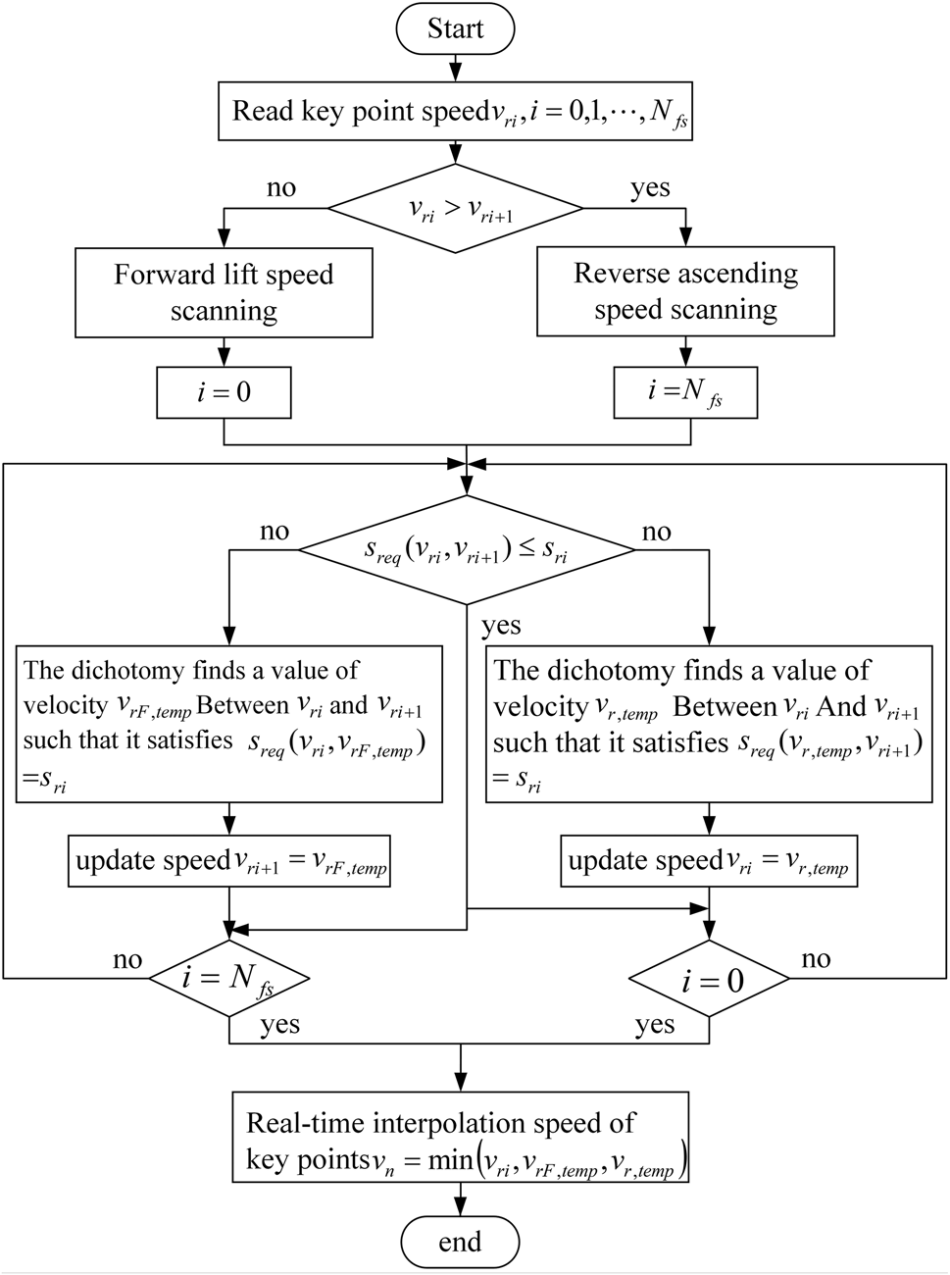
Update flow chart of bidirectional scanning speed of key points.

According to Eq. (21), the arc lengths of curves between key points are calculated. Section 2.1 only determines the velocity of key points according to geometric and kinematic constraints. This method can only ensure that the speed at the key points will not cause the chord error to exceed the limit and that the speed, acceleration and elevated acceleration are limited within the given range. Also, we need to consider the curves’ arc length according to the key point of piecewise whether can satisfy the required arc length of the acceleration and deceleration during the real-time interpolation. If the actual arc length between key points cannot meet the required arc length, then the velocity of each key point needs to be further updated to ensure that the overall velocity of the curve after the segmentation at each key point is smooth. Therefore, to improve the speed updating efficiency at key points, a speed scanning with forward and reverse bidirectional is adopted to further update the speed at key points according to the arc length of each sub-segment. The scanning process is shown in Fig. 5.

Let *i* = 0 represent the initial key point, that is, the beginning of the curve; $i = N_{fs}$ represent the end key point, that is, the end of the curve; $v_{ri}$ is the speed of the previous key point; $v_{ri + 1}$ is the speed of the next key point. In reverse scanning, the first set $i = N_{fs}$, if $v_{ri}$ is greater than $v_{ri + 1}$, it is the process of deceleration; otherwise, is the process of acceleration. Then, the curve arc length *S* between the $ri$ and $ri + 1$ key points is compared with the smaller arc length $s_{req} \left( {v_{ri} ,v_{ri + 1} } \right)$ needed by the previous key point velocity $v_{ri}$ to the next key point velocity $v_{ri + 1}$ according to Eq. (20), if $s_{req} \left( {v_{ri} ,v_{ri + 1} } \right) \le s_{ri}$, then the arc length of this section can complete the deceleration process; otherwise, the deceleration process cannot be realized. Then, the dichotomy method is adopted to select velocity $v_{r,temp}$ between velocity $v_{ri}$ and $v_{ri + 1}$ so that it meets $s_{req} (v_{r,temp} ,v_{ri + 1} ) = s_{ri}$, the speed at this key point is updated to $v_{r,temp}$, so that it meets the deceleration process, and the above process is repeated until the deceleration reverse tracing process is finished at $i = 0$; In forward scanning, the value starts from $i = 0$, if $v_{ri} < v_{ri + 1}$, the speed increases. Then, the curve arc length $s_{ri}$ between key points $ri$ and $ri + 1$ is compared with the arc length $s_{req} \left( {v_{ri} ,v_{ri + 1} } \right)$, required from the previous key point speed of $v_{ri}$ to the next key point speed of $v_{ri + 1}$, according to Eq. (20). If $s_{req} \left( {v_{ri} ,v_{ri + 1} } \right) \le s_{ri}$, the arc length of this section can complete the speed increase process; otherwise, the speed increase process cannot be realized. The dichotomy method is adopted to select the speed $v_{rF,temp}$ between the speed $v_{ri}$ and the speed $v_{ri + 1}$ so that it meets $s_{req} (v_{ri} ,v_{rF,temp} ) = s_{ri}$, and the speed at this key point is updated to $v_{rF,temp}$ so that it meets the speed decrease process. The above process is repeated until the speed decrease reverse scanning process is completed at $i = 0$. Ultimately, the key point federate $v_{i}$ is taken to be the minimum value after the forward and reverse bi-directional sweep update.

22 $$v_{i} = \min \left( {v_{ri} ,v_{rF,temp} ,v_{r,temp} } \right)$$

where $v_{ri}$ is the nominal speed, $v_{rF,temp}$ is the forward scanning speed, $v_{r,temp}$ is the reverse scanning speed.

After forward and reverse speed scanning update, the speed values at each key point can be further restricted within the constraint range. The key points and their allowable speed values are obtained. The planned feed speed adopts the constrained speed value at key points, and at non-key points, the speed changes smoothly according to the S-type acceleration and deceleration method planned in Section 2.2.

### Real time interpolation

After the feeding speed planning was completed, the Sect. 1.2 interpolation parameter calculation method was adopted to sample with a fixed period T based on the S-type acceleration and deceleration planning, calculate the arc length increment of the current interpolation cycle $\Delta s$, to further determine the next interpolation parameter $u$, and finally determine the interpolation trajectory curve.

## Results and discussion

**Figure 6:**
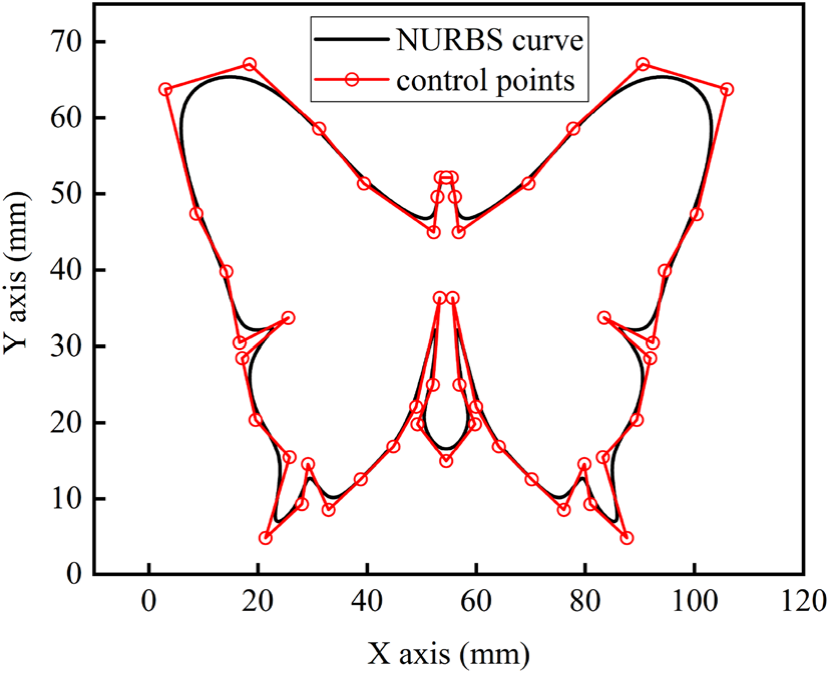
NURBS curve.

In order to verify the effectiveness of the algorithm proposed in this paper, MATLAB software was used to simulate a cubic NURBS curve with 51 control points as shown in Fig. 6. At the same time, set simulation parameters as shown in Table 1.

**Table 1:** Simulation interpolation parameter.

Parameter	Numerical value
Interpolation period $T{\text{/ms}}$	1
Programming feed speed $v_{p} {\text{/(mm/s)}}$	100
Chord error limit $\delta_{\lim } {/}\upmu {\text{m}}$	1
Normal acceleration limit $a_{n\max } {\text{/(mm/s}}^{{2}} {)}$	1000
Normal jerk limit $J_{a\max } {\text{/(mm/s}}^{{3}} {)}$	$1 \times 10^{4}$

**Figure 7:**
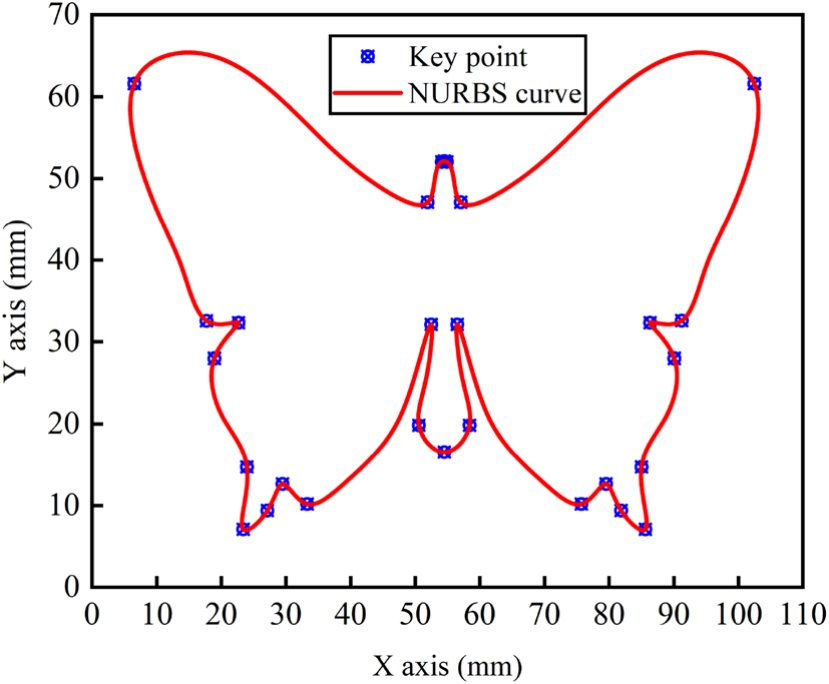
Key point.

**Figure 8:**
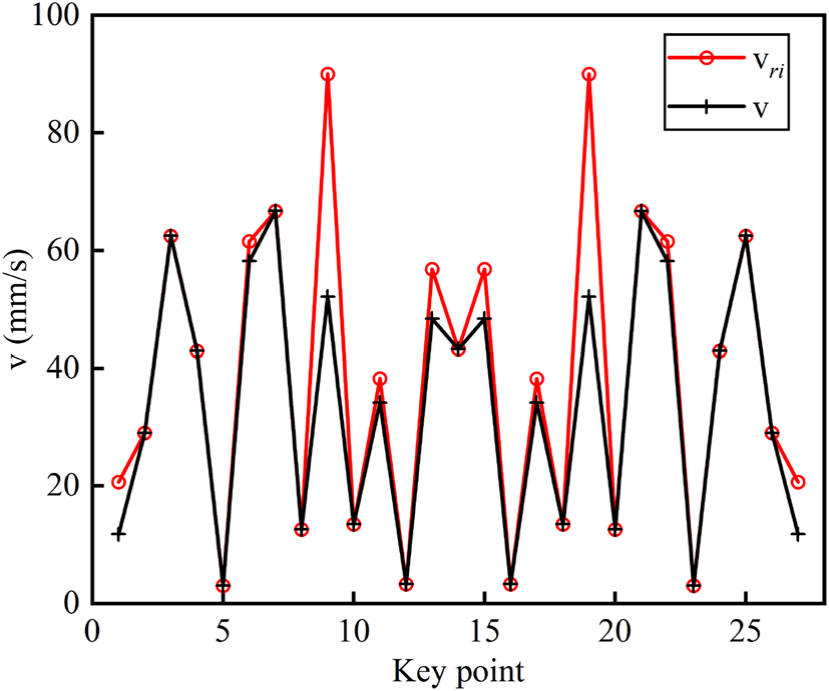
Key point speed update.

In the pre-processing stage, the velocity planning algorithm will first obtain the curvature threshold according to the constraints, then search to get all the key points and segment the NURBS curve at the key points, and finally update the key point velocities by S-type acceleration/ deceleration methods and forward scanning of the speed-up process and reverse scanning of the speed-down process to ensure the global light smoothness of the velocities. According to the simulation parameters set in Table 2 and Eq. (21), the threshold value of curvature is calculated to be $\kappa_{0} = 70.7\,\upmu {\text{m}}^{ - 1}$, with a total of 28 key points and 27 sub-segments, as shown in Figs. 7, 8 shows the nominal speed at each key point and the speed after a bidirectional scanning update.

**Table 2:** Calculation comparison results of each interpolation algorithm.

Interpolation method	Maximum feed speed fluctuation rate (%)	Calculation time ($\upmu {\text{s}}$)
First-order Taylor expansion	6.84	3.9
Fourth-order Runge–Kutta method	0.36	16.1
Methodology of this article	0.0281	11.5

**Figure 9:**
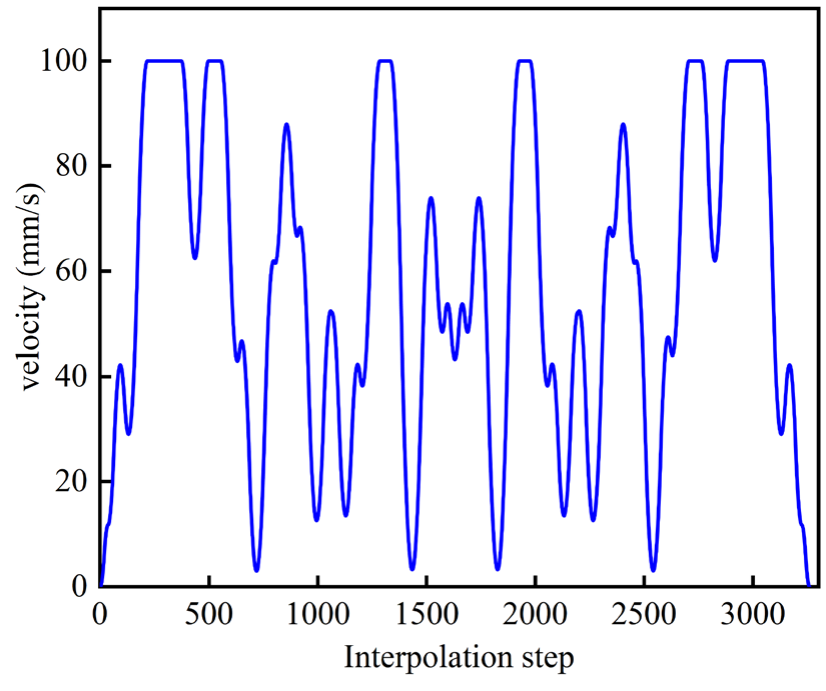
Feed speed curve.

**Figure 10:**
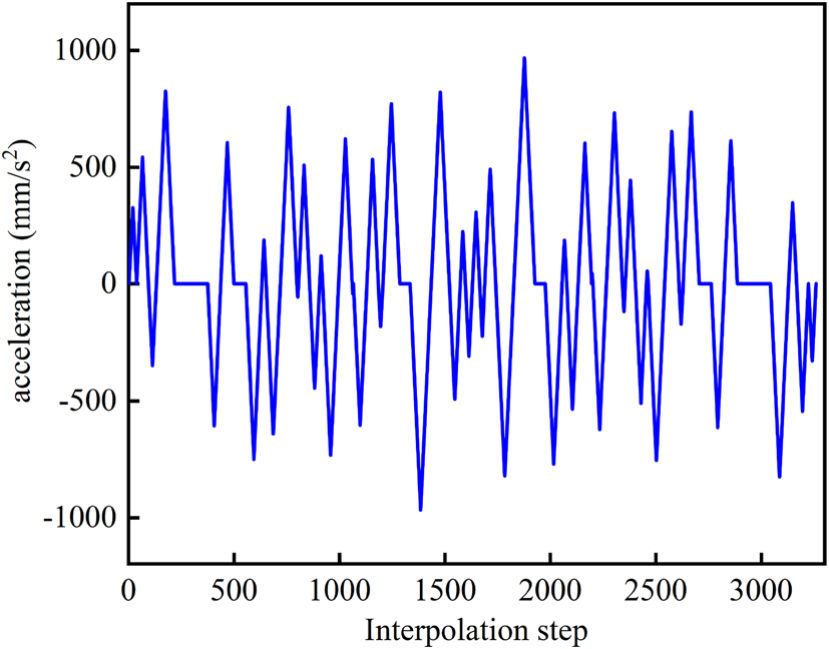
Acceleration curve.

**Figure 11:**
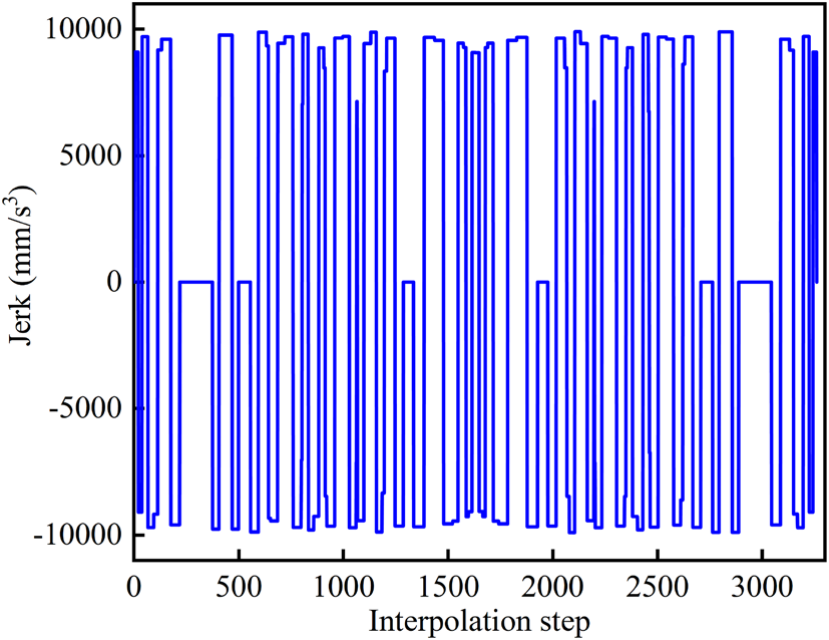
Jerk curve.

**Figure 12:**
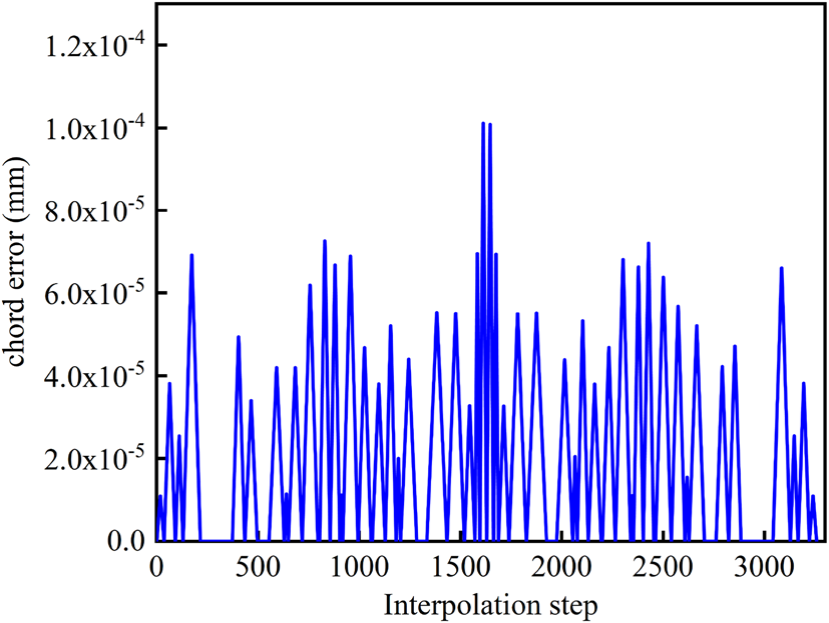
Chord error.

The velocity planning method proposed in this paper is used to obtain the feed velocity, normal acceleration, and normal jerk curves of the overall curve in the process of interpolation, as shown in Figs. 9, 10 and 11. Each component is well restricted within the set range. It can be seen from Fig. 11 that the jerk corresponding to the velocity planning method obtained a maximum value at multiple positions. Figure 12 shows the chord error curve in the interpolation process, and it can be seen that the processing error is completely limited within the allowable range of processing.

**Figure 13:**
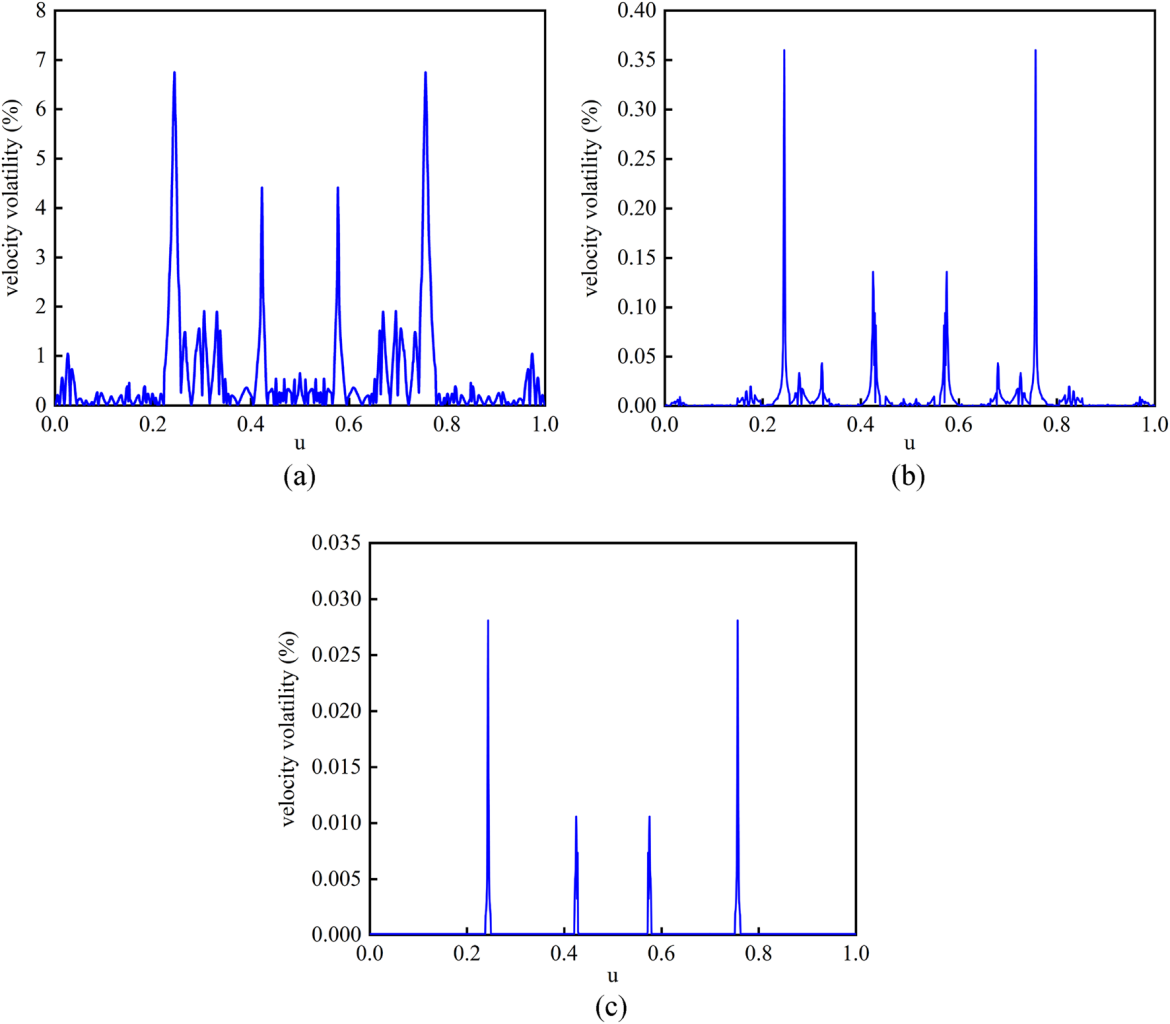
Feed velocity fluctuation of three interpolation algorithms (a) First-order Taylor expansion method, (**b**) Fourth-order Runge–Kutta method, (c) Parameter-corrected second-order Runge–Kutta method.

To further verify the advantages of the piecewise bidirectional NURBS curve speed update method based on key points and the second-order Runge–Kutta interpolation algorithm with parameter modification proposed in this paper, the simulation results were compared with the first-order Taylor expansion method and the fourth-order Runge–Kutta method, which also only need to calculate the NURBS first-order derivative. The feed velocity volatility at each interpolation point was calculated $\delta_{i}$^32^. The feed velocity volatility of the three methods was shown in Fig. 13, and the calculation time comparison was shown in Table 2.

The first-order Taylor expansion method only needs the first-order derivative once in the calculation of interpolation parameters, which has the shortest calculation time. However, due to the large truncation error, the velocity fluctuation rate is the largest. The fourth-order Runge–Kutta method requires a total of four first-order derivative operations, and the calculation time is the longest, and the calculation accuracy is higher and the velocity fluctuation is smaller compared with the first-order Taylor expansion method. The method adopted in this paper requires a total of three first-order derivative calculations, including two first-order derivative calculations and one derivative calculation when calculating the correction of parameters, so the velocity fluctuation is the smallest, the interpolation parameter calculation time is smaller than the fourth-order Runge–Kutta method and larger than the first-order Taylor expansion method, and the interpolation accuracy is higher.

## Conclusion

In this paper, a NURBS interpolation algorithm with arc length segmentation is proposed. According to geometric constraints and kinematic constraints, the curvature threshold is calculated to obtain the nominal speed at the key points, NURBS curves are segmented at the key points, and the arc lengths of each segment are calculated by the adaptive Simpson method. Based on the S-shaped speed planning method, the speed at the key points is updated by bidirectional scanning, and the global smoothing of the speed is realized. In the stage of real-time interpolation, to improve the real-time performance of interpolation calculation, the second-order Runge–Kutta method with parameter correction is used to calculate the parameters of the real-time interpolation curve, which can effectively reduce the amount of computation in interpolation and keep high interpolation accuracy. The interpolation simulation verifies that the proposed velocity planning method and the interpolation parameter calculation method can take good care of the interpolation accuracy and interpolation real-time performance.

## Data Availability

The datasets used or analyzed during the current study are available from the corresponding author on reasonable request.
